# Integrative Multi-Omics Analysis of Gill Responses to Long-Term Salinity Stress in Grass Carp (*Ctenopharyngodon idella*)

**DOI:** 10.3390/antiox15091070

**Published:** 2026-08-26

**Authors:** Linjun Zhou, Xiajie Chen, Yiran Hou, Chengfeng Zhang, Jian Zhu, Bing Li, Rui Jia

**Affiliations:** 1 Key Laboratory of Integrated Rice-Fish Farming Ecology, Ministry of Agriculture and Rural Affairs, Freshwater Fisheries Research Center, Chinese Academy of Fishery Sciences, Wuxi 214081, China; zhoulinjun@ffrc.cn (L.Z.);; 2 Wuxi Fisheries College, Nanjing Agricultural University, Wuxi 214081, China

**Keywords:** salinity stress, multi-omics, ion regulation, PPAR signaling pathway, calcium signaling pathway, *Ctenopharyngodon idella*

## Abstract

Salinity is an important environmental factor affecting the physiological homeostasis of freshwater fish, yet the underlying mechanisms in grass carp (*Ctenopharyngodon idella*) gills remain unclear. Therefore, grass carp were exposed to different salinity levels for 60 days, and gill responses were evaluated using histopathological, ion regulatory, antioxidant, transcriptomic, and metabolomic analyses. Histological observations showed that high salinity (8 g/L) caused marked structural damage to the gill lamellae. Specifically, Na^+^ and Ca^2+^ concentrations and Na^+^/K^+^-ATPase activity significantly decreased, while K^+^ concentration and Ca^2+^-ATPase activity increased, revealing disrupted ion homeostasis. Salinity exposure also led to decreased antioxidant enzyme activities. Integrated omics analysis further demonstrated that a total of 2447 differentially expressed genes and 268 differentially expressed metabolites were identified, with significant enrichment in pathways related to biosynthesis of amino acids, arachidonic acid metabolism, glutathione metabolism, PPAR signaling, and calcium signaling. Notably, the PPAR and calcium signaling pathways showed positive enrichment under salinity stress, suggesting their potential involvement in the regulation of lipid metabolism, energy allocation, and cellular stress responses. Our findings indicated amino acid biosynthesis and arachidonic acid metabolism as key pathways involved in the adaptation of grass carp gills to salinity stress. Overall, chronic salinity exposure caused structural alterations, disrupted ion regulation, altered antioxidant status, and marked transcriptomic and metabolomic changes in grass carp gills, offering new insight into salinity adaptation in freshwater fish.

## 1. Introduction

Saline–alkaline environments are widely distributed and pose major challenges to agricultural and aquaculture production. China possesses abundant saline–alkaline water resources, with an estimated area of approximately 690 million mu (about 46 million hectares) [1]. Despite their considerable potential for aquaculture development, these resources remain largely underutilized. In recent years, saline–alkaline water aquaculture has emerged as a promising strategy for resource utilization, with the potential to improve water quality and generate substantial economic benefits. Accordingly, increasing efforts have been made to evaluate the suitability of aquatic species for farming in saline–alkaline environments, including tilapia (*Oreochromis* spp.) [2], channel catfish (*Ictalurus punctatus*) [3], and Amur ide (*Leuciscus waleckii*) [4]. However, the further development of this industry is still constrained by limited understanding of the physiological and metabolic responses of many candidate species to saline–alkaline stress.

Environmental salinity is a major ecological factor that directly alters the osmotic gradient between fish body fluids and the surrounding water, thereby affecting water and ion balance [5]. To maintain internal osmotic and ionic homeostasis, fish must dynamically regulate ion transport, osmolyte composition, and associated ATP-dependent processes. Accordingly, salinity exposure commonly induces changes in plasma osmolality and ionic composition [6]. For instance, acute exposure to high salinity (20 ppt) significantly increased serum osmolality in Acipenser sinensis, although this response declined with prolonged exposure [7]. Similarly, salinity exposure (5–14 ppt) markedly elevated plasma Na^+^ and Cl^−^ concentrations as well as osmotic pressure in *Luciobarbus capito* [8]. In addition, the activity of key ion-transport enzymes such as Na^+^/K^+^-ATPase can be altered during salinity acclimation, reflecting the physiological adjustments required to maintain ionic balance under osmotic challenge [5]. Consistent with this, salinity exposure (2–8 ppt) increased Na^+^/K^+^-ATPase activity in *Oreochromis niloticus* [9]. Osmoregulation is energetically demanding and is therefore closely linked to intermediary metabolism. Energy allocated to ion transport and cellular homeostasis may reduce the energy available for other physiological processes, including somatic growth and reproduction [10]. Consistent with this concept, in common carp, rising salinity reduced food consumption and growth rate while increasing oxygen consumption [11]. To meet the additional energetic requirements imposed by osmotic stress, fish can reorganize carbohydrate, lipid, and amino acid metabolism, thereby altering energy production, substrate utilization, and metabolic resource allocation. Previous studies have shown that salinity exposure affects key pathways involved in glycolysis/gluconeogenesis, the tricarboxylic acid cycle, and mitochondrial oxidative phosphorylation [12,13]. Salinity stress may also induce oxidative stress and inflammation, as reflected by increased antioxidant enzyme activities and inflammatory cytokine levels in *Notopterus chitala* [14]. In addition, salinity exposure significantly increased reactive oxygen species (ROS) levels and caused oxidative damage in the blood cells of Nile tilapia (*Oreochromis niloticus*) [15]. Thus, the response to salinity involves an integrated adjustment of osmoregulatory, metabolic, and antioxidant processes rather than changes in ion regulation alone.

The fish gill is a critical interface between the organism and the aquatic environment, mediating essential physiological functions such as respiration, osmoregulation, nitrogenous waste excretion, and immune defense [16]. This direct exposure also makes the gill a primary site for sensing environmental fluctuations and mediating subsequent stress responses. It exhibits significant plasticity, rapidly adapting its morphology, cellular composition, and gene expression to physicochemical changes (e.g., temperature, salinity, pH) and biological challenges like pathogens [17,18]. Among these stressors, salinity variation has been widely recognized as a key factor affecting gill structure and function. For example, in *Oreochromis niloticus*, high salinity under both direct and gradual acclimation altered branchial Na^+^/K^+^-ATPase activity and chloride cell abundance [19]. Similarly, transfer of Persian sturgeon (*Acipenser persicus*) from freshwater to brackish water (11‰) induced significant structural alterations in the gills [20].

The grass carp (*Ctenopharyngodon idella*) is one of the most important freshwater economic fish species in China and has one of the highest production volumes among farmed freshwater fishes. Although generally regarded as a freshwater species with limited salinity tolerance, increasing evidence suggests that grass carp can tolerate saline–alkaline environments to some extent, highlighting its potential for saline–alkaline aquaculture. Previous studies have begun to explore the responses of this species to salinity stress. The 96 h half-lethal salinity concentration for grass carp was determined to be 11.73‰, but in another study, grass carp have been maintained at salinities of up to 12 g/L for three weeks [21,22,23]. In addition, moderate salinity (0–6 ppt) has been reported to improve muscle quality in grass carp [24], whereas exposure to 6 ppt caused growth inhibition and significant physiological and immune disturbances [21]. Acute high-salinity stress (10 ppt) also induced marked elevations in serum electrolytes and cortisol [25]. Although integrated transcriptomic and metabolomic approaches have been increasingly applied to investigate salinity responses in teleost fish [26,27], these studies have demonstrated that the use of multi-omics approaches alone does not fully resolve how tissue-level physiological alterations are linked to molecular and metabolic responses during prolonged salinity exposure. In grass carp, particularly, the relationships among gill structural alterations, local ion regulation, redox homeostasis, and intermediary metabolic reprogramming under long-term salinity exposure remain insufficiently characterized. Therefore, this study integrated histopathological, physiological, transcriptomic, and metabolomic analyses to investigate the mechanisms underlying gill responses to salinity stress in grass carp, and to identify signaling pathways involved in salinity adaptation. These findings are essential for advancing the application and genetic improvement of grass carp in saline–alkaline aquaculture.

## 2. Materials and Methods

### 2.1. Experimental Design

Juvenile grass carp (body weight, 100.0 ± 5.0 g) were sourced from the experimental farm of FFRC (Freshwater Fisheries Research Centre, Wuxi, China). A two-week acclimation period was conducted in a recirculating aquaculture system before the experiment. The fish received twice-daily feedings of a commercial diet (Tongwei, Chengdu, China) at a ratio of 2% of their body weight during acclimation. The water quality was controlled and maintained at the following levels: temperature, 28.0 ± 2.0 °C; pH, 6.8–7.6; dissolved oxygen, >6 mg/L.

Following acclimation, all fish were pooled, and individuals of similar body size were randomly captured and sequentially assigned to nine experimental tanks until each tank contained 12 fish. These groups were exposed to salinities of 0 g/L (normal control, NC), 4 g/L, and 8 g/L, respectively. Each treatment group, consisting of 36 fish, was maintained in three replicate tanks (12 fish per tank). The internal dimensions of each tank were 120 × 60 × 40 cm, and the water level was maintained at approximately 260 L. The initial stocking density was approximately 4.6 g/L. Each tank had an independent water supply and drainage system and was equipped with a separate commercial external filter. Before the formal exposure period, salinity was gradually increased at approximately 1 ppt day^−1^ until the designated salinity of each treatment was reached. The 60-day exposure period commenced only after all groups had reached their respective target salinities. Salinity was monitored daily using a handheld salinity meter (WS-31plus, Ruishui, Zhejiang, China; measurement range: 0–55 ppt; resolution: 0.1 ppt) after the routine water exchange. To maintain water quality, approximately 20% of the water in each tank was replaced daily with water that had been pre-adjusted to the corresponding treatment salinity. During the 60-day exposure period, fish were maintained under a 12 h light:12 h dark photoperiod and were fed a commercial diet (Tongwei, Chengdu, China) twice daily at approximately 2% of the total biomass. After 60 days of exposure, fish in the 8 g/L group exhibited a significantly lower final body weight than those in the 0 and 4 g/L groups (*p* < 0.05). Survival was 100% in both the 0 and 4 g/L groups and 97.2% in the 8 g/L group. Detailed final body-weight and survival data are provided in Supplementary Table S1.

After the 60-day exposure period, fish were fasted for 24 h before sampling. Four fish were randomly sampled from each tank and anesthetized with 50 mg/L MS-222. Gill tissues were immediately excised from each fish and allocated for the different analyses. A portion of the gill tissue was fixed in 4% paraformaldehyde for histological examination, whereas the remaining tissue was immediately frozen in liquid nitrogen and stored at −80 °C for biochemical, transcriptomic, metabolomic, and qPCR analyses. Physiological and biochemical measurements were performed separately on individual fish samples, resulting in 12 individual fish measurements per treatment. For transcriptomic and metabolomic analyses, equal amounts of frozen gill tissue from the four fish within each tank were pooled to generate one tank-level composite sample. The experimental protocol was approved by the Freshwater Fisheries Research Center, Chinese Academy of Fishery Sciences. All procedures involving experimental fish were carried out in accordance with institutional guidelines and national regulations for animal welfare and the ethical use of laboratory animals.

### 2.2. Histological Analysis of Gill Tissue

Gill tissues were fixed in 4% paraformaldehyde for 24 h, dehydrated in graded ethanol, cleared in xylene, and embedded in paraffin. Sections (4–5 μm) were prepared using a rotary microtome, mounted on glass slides, deparaffinized, and rehydrated. The sections were stained with hematoxylin and eosin (H&E) following standard procedures, dehydrated, cleared, and sealed with neutral resin. Histological changes were examined under a light microscope. Histological examination was performed using three gill tissue sections per treatment, with each section obtained from a different fish. To improve comparability among individuals, gill tissues were sampled from comparable anatomical regions. One section from each fish was examined, and approximately three non-overlapping microscopic fields were evaluated per section. Histological observations were performed under blinded conditions, with treatment identity concealed during microscopic examination.

### 2.3. Ion Concentration and Enzyme Activity Assay

Gill tissues (approximately 100 mg) were homogenized in nine volumes of deionized water and centrifuged at approximately 700× *g* for 10 min at 4 °C. The resulting supernatant was collected for the determination of Na^+^ (C002-2-1), K^+^ (C001-2-1), Cl^−^ (C003-2-1), and Ca^2+^ (C004-2-1) concentrations using commercial assay kits (Nanjing Jiancheng Bioengineering Institute, Nanjing, China) in accordance with the manufacturer’s instructions. Ion concentrations were normalized to the wet weight of the gill tissue and expressed as mmol g^−1^ wet gill tissue. Na^+^/K^+^-ATPase activity and Ca^2+^-ATPase activity were determined according to the method described by published research studies [28,29,30]. The detailed description of the methods is provided in the Supplementary Materials (Method S1 and S2).

### 2.4. Measurement of Gill Oxidative Stress Parameters

Gill tissues were homogenized on ice with cold physiological saline at a ratio of 1:9 (*w*/*v*). The homogenate was then centrifuged at 3600 rpm for 10 min at 4 °C. The resulting supernatant was collected and used to assay oxidative stress parameters, including superoxide dismutase (SOD), glutathione (GSH), total antioxidant capacity (T-AOC), glutathione peroxidase (GPx), malondialdehyde (MDA), and protein carbonyl levels. SOD activity was determined using the water-soluble tetrazolium salt (WST-1) method [31]. GPx activity was measured with cumene hydroperoxide as the substrate and 5,5′-dithiobis(2-nitrobenzoic acid) (DTNB) as the chromogen [32]. The GSH content was assessed by measuring the yellow derivative produced in its reaction with DTNB [33]. T-AOC was evaluated using the ferric reducing ability of plasma (FRAP) assay. MDA concentration was assessed by the thiobarbituric acid (TBA) method [34]. Protein carbonyl content was determined separately using the 2,4-dinitrophenylhydrazine (DNPH) derivatization method (see Method S3 for details). The protein content in the gill tissue was measured using the Bicinchoninic Acid (BCA) method. Assay kits for SOD (A001-3-2), T-AOC (A015-2-1), GSH (A006-2-1), and protein carbonyl (A087-1-2) were purchased from the Nanjing Jiancheng Bioengineering Institute (Nanjing, China). The MDA assay kit (S0131S) was obtained from Beyotime Biotechnology (Nantong, China), and the GPx assay kit (G0204W48) was acquired from Grace Biotech (Suzhou, China). All parameters were measured strictly following the manufacturers’ instructions.

### 2.5. Metabolomics Analysis in Gills

Approximately 100 mg of each gill sample was homogenized in 1 mL of a pre-chilled extraction solution (methanol: acetonitrile: water = 2:2:1, *v*/*v*/*v*). The homogenate was centrifuged at 13,000× *g* for 15 min at 4 °C. The resulting supernatant was collected and vacuum-dried into a lyophilized powder. The dried residue was then reconstituted in 100 μL of a 50% acetonitrile-water solution (*v*/*v*). After another centrifugation at 14,000× *g* for 15 min at 4 °C, the supernatant was subjected to LC–MS analysis. The analysis was performed on an ultra-high-performance liquid chromatography (UHPLC) system (1290 Infinity LC, Agilent Technologies, Santa Clara, CA, USA) coupled with a quadrupole time-of-flight mass spectrometer (TripleTOF 6600, AB Sciex, Marlborough, MA, USA). Detailed procedures are provided in Supplementary Method S4.

Following conversion to mzML format using ProteoWizard (v3.0.6428), the raw data were processed with the online XCMS platform (v3.7.1) for feature extraction. Subsequent steps included metabolite identification, data preprocessing, and final data analysis (Method S5). To ensure the reliability and quality of the metabolomic data, quality control (QC) samples were analyzed throughout the acquisition process for monitoring and assurance. Orthogonal partial least squares-discriminant analysis (OPLS-DA) was conducted using the ropls package in R (Version 4.2.2). The model’s validity was evaluated by cross-validation and permutation tests. Significantly altered metabolites between comparison groups were screened by combining the variable importance in projection (VIP) scores from the OPLS-DA model with *p*-values derived from Student’s *t*-test, using a threshold of VIP ≥ 1 and *p* < 0.05. Kyoto Encyclopedia of Genes and Genomes (KEGG) pathway enrichment analysis was then performed on the annotated differential metabolites to reveal the key metabolic and signal transduction pathways involved.

### 2.6. Transcriptome Sequencing and Analysis

For transcriptomic analysis, equal amounts of frozen gill tissue from the four fish sampled within each tank were pooled to generate one tank-level composite sample. Pooling was performed only within each tank, with no mixing of tissues among tanks. Thus, three independent tank-level composite biological samples were generated per treatment, corresponding to the three replicate tanks. Transcriptomic sequencing was performed for the control (CG, 0 g/L) and high-salinity (SG, 8 g/L) groups, resulting in six RNA-seq libraries in total. Total RNA from gill tissues was extracted using TRIzol reagent (Invitrogen, Carlsbad, CA, USA) according to the manufacturer’s protocol. RNA quality was verified using the Agilent 2100 Bioanalyzer for assessment and agarose gel electrophoresis for visual confirmation. Following quality assessment, mRNA was enriched from total RNA using mRNA Capture Beads. The purified mRNA was subsequently fragmented and used as a template for cDNA synthesis. The resulting cDNA was end-repaired, ligated with adaptors, and amplified by PCR to construct the sequencing library. The final library was sequenced on an Illumina NovaSeq X Plus platform (Guangzhou Gidio Biotechnology, Guangzhou, China).

The raw sequencing data were processed with fastp to obtain high-quality clean reads [35]. Subsequently, the clean reads were aligned to the species-specific ribosomal RNA (rRNA) database using Bowtie2 to identify and remove rRNA-derived reads [36]. The resulting rRNA-filtered reads were then aligned to the reference genome (NCBI accession: GCF_019924925.1) using HISAT2 [37]. The transcriptome was assembled using StringTie, which facilitated the annotation of novel genes. Gene expression abundance was expressed as fragments per kilobase of transcript per million mapped reads (FPKM) for descriptive purposes [38,39]. Principal component analysis (PCA) was performed to assess inter-sample relationships. Intergroup comparisons with biological replicates were performed using DESeq2 (v1.24), with raw counts normalized before model-based hypothesis testing, *p* values adjusted by the false-discovery-rate procedure, and genes defined as differentially expressed when FDR < 0.05 and |log_2_FC| > 1 [40]. These DEGs were subsequently subjected to functional enrichment analysis using Gene Ontology (GO) and KEGG databases. Furthermore, Gene Set Enrichment Analysis (GSEA) was applied to the entire transcriptome dataset [41]. Gene sets with a normalized enrichment score (NES) absolute value greater than 1, a nominal *p* (NOM *p*-val) less than 0.05, and an FDR value less than 0.25 were considered statistically significant. The raw sequencing data have been deposited in the Genome Sequence Archive (GSA) of the China National Center for Bioinformation (CRA038997). In addition, selected differentially expressed genes identified from the transcriptomic analysis were further validated by quantitative real-time PCR (qPCR) and the specific primers used are provided in Table S2. Detailed procedural steps are described in Supplementary Method S6.

### 2.7. Statistical Analysis

The data were analyzed using SPSS Statistics software (version 26.0). After verification of normality using the Shapiro–Wilk test and homogeneity of variances using Levene’s test, the effects of salinity on ion concentrations and oxidative stress parameters were evaluated by one-way analysis of variance (ANOVA), followed by least significant difference (LSD) post hoc tests. Differences in gene expression between the control (CG) and 8 g/L salinity (SG) groups were analyzed using an independent-samples *t*-test. Multivariate statistical analyses were additionally performed for the omics datasets. Principal component analysis (PCA) was used for both metabolomic and transcriptomic datasets to evaluate overall variation among samples and visualize separation between the control and salinity-treated groups. For the metabolomic dataset, orthogonal partial least squares-discriminant analysis (OPLS-DA) was performed using the ropls package in R, and model robustness was evaluated by cross-validation and permutation testing. The correlation between RNA-seq and qPCR results was assessed using Pearson’s correlation analysis. *p* < 0.05 was considered statistically significant.

## 3. Results

### 3.1. Histopathological Alterations in Gills

**Figure 1:**
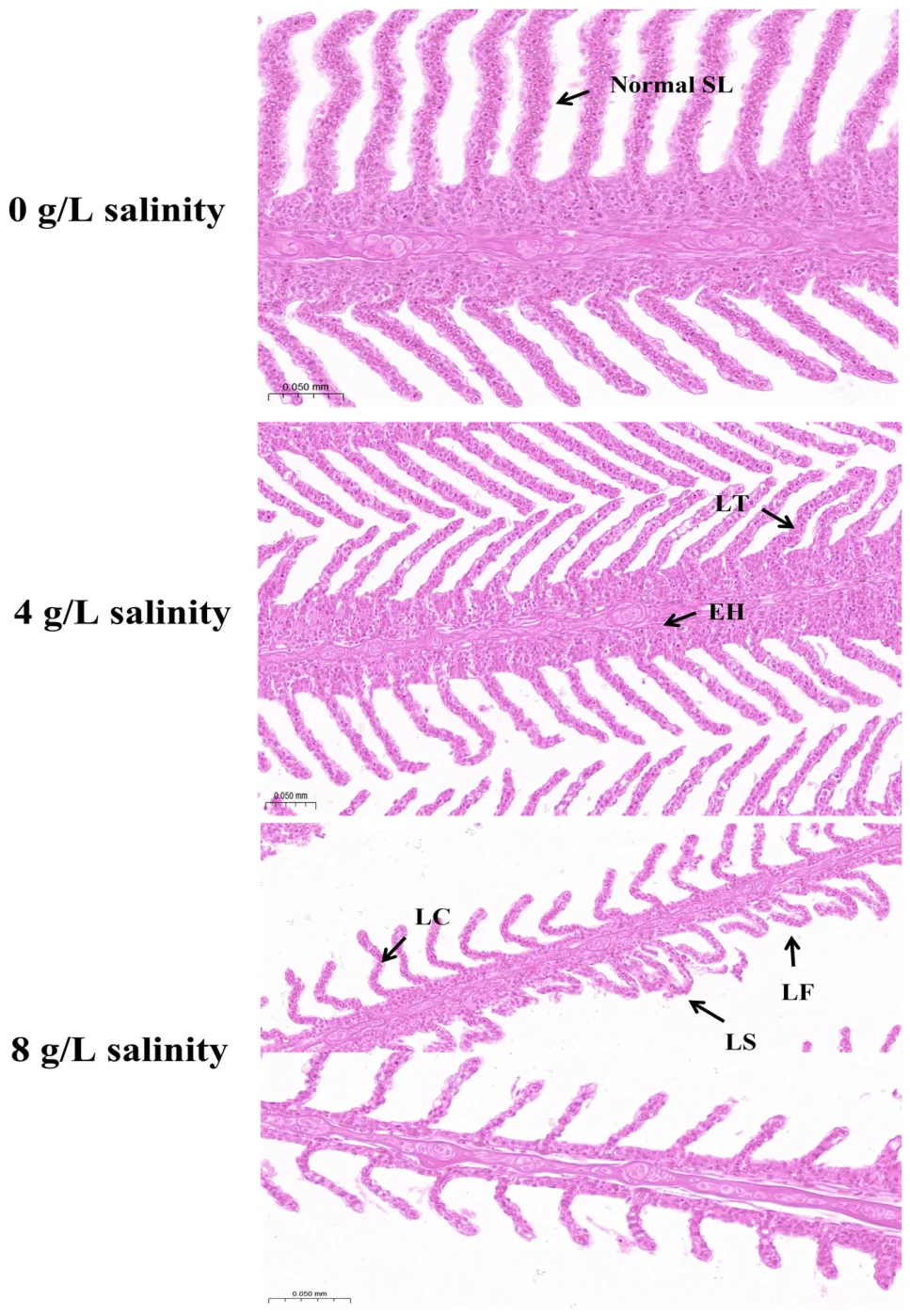
Histopathological analysis of gill tissues after salinity exposure. Representative hematoxylin and eosin (H&E)-stained sections are shown for the 0, 4, and 8 g/L salinity groups. Gill architecture was generally intact in the 0 g/L group. Mild epithelial hyperplasia (EH) and lamellar thickening (LT) were observed at 4 g/L, whereas the 8 g/L group exhibited more pronounced alterations, including lamellar shortening (LS), lamellar curvature (LC), lamellar fusion (LF), and epithelial thickening. Arrows and arrowheads indicate the representative histopathological features, and enlarged views show the corresponding lesions at higher magnification. Scale bars are indicated in the individual panels. Bar = 0.05 mm.

As salinity increased, progressively more pronounced structural alterations were observed in the gills of grass carp (Figure 1). At 4 g/L salinity, the gill lamellae exhibited mild epithelial hyperplasia and structural thickening, which may reflect compensatory tissue remodeling in response to moderate salinity exposure. At 8 g/L salinity, the lamellae were markedly shortened, curved, and fused, accompanied by pronounced epithelial thickening, indicating more substantial structural disturbance under elevated salinity exposure.

### 3.2. Changes in Ion Concentrations in Gills

**Figure 2:**
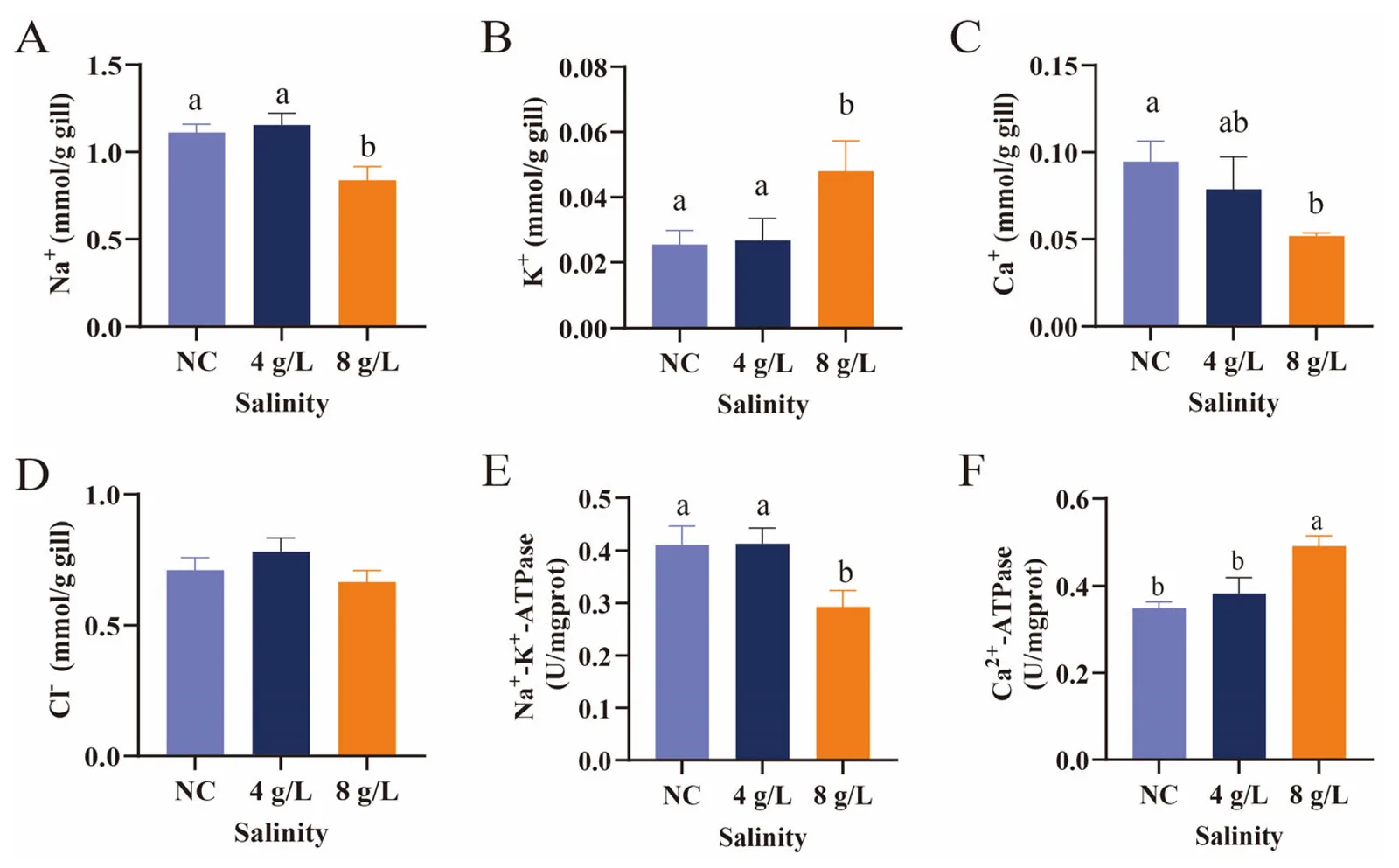
Changes in ion concentrations and ion-regulatory enzyme activities in the gills of grass carp. (**A**) Na^+^, (**B**) K^+^, (**C**) Ca^2+^, (**D**) Cl^−^, (**E**) Na^+^/K^+^-ATPase, and (**F**) Ca^2+^-ATPase. Data are expressed as mean ± SEM based on 12 individual fish measurements per treatment, comprising four fish sampled from each of three replicate tanks. Different letters above the bars indicate significant differences among groups at *p* < 0.05.

After 60 days of salinity exposure, significant alterations in ion concentrations were observed in the 8 g/L group. Specifically, Na^+^ and Ca^2+^ concentrations decreased significantly, whereas K^+^ concentration increased significantly (*p* < 0.05; Figure 2A–C). In addition, Na^+^/K^+^-ATPase activity was significantly reduced, while Ca^2+^-ATPase activity was significantly increased in the 8 g/L group (*p* < 0.05; Figure 2E,F). In contrast, the 4 g/L treatment caused no significant changes in ion concentrations or ion regulatory enzyme activities.

### 3.3. Variations in Antioxidant Status in Gills

**Figure 3:**
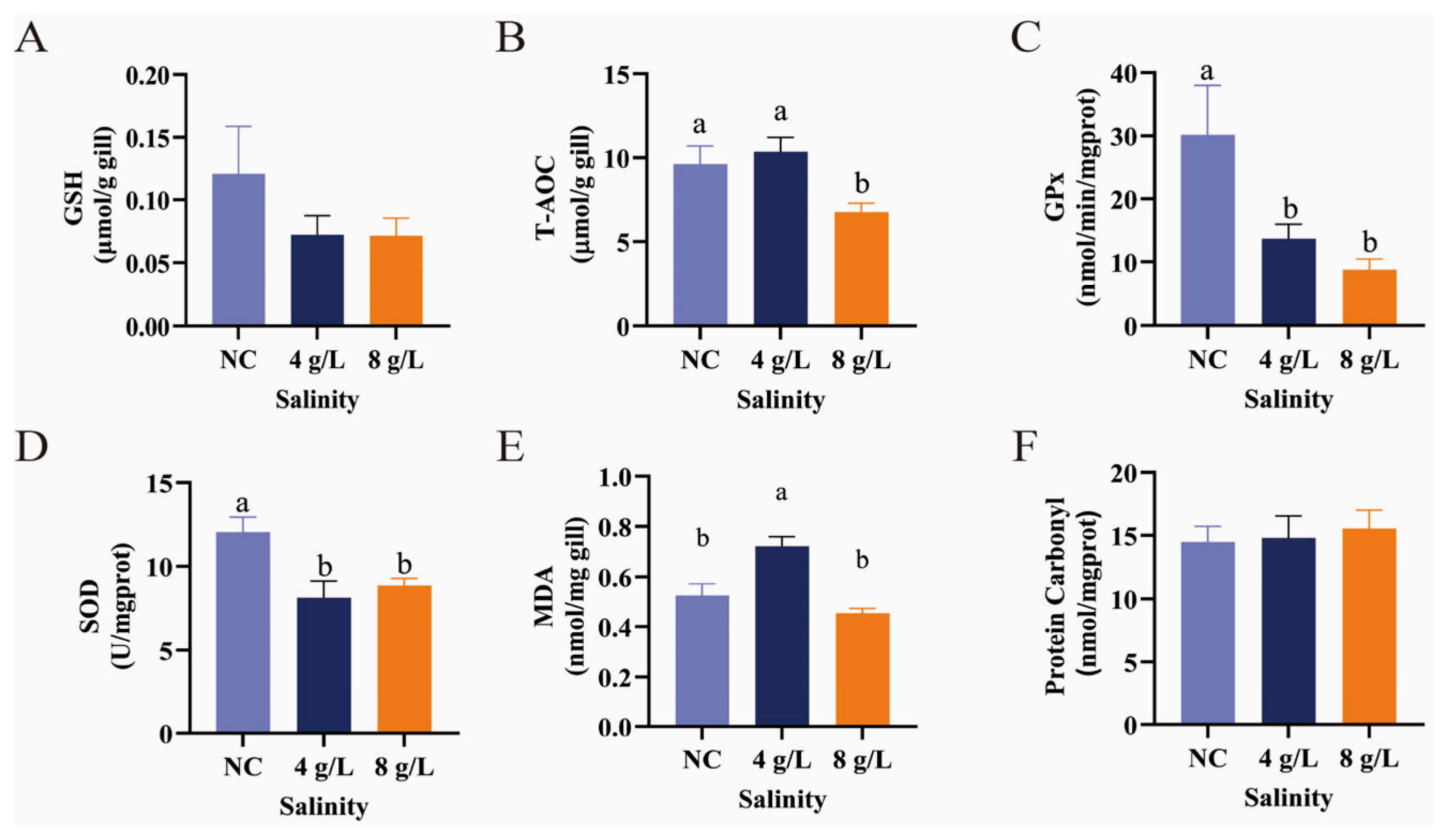
Changes in antioxidant status in the gills of grass carp under salinity exposure. (**A**) GSH, (**B**) T-AOC, (**C**) GPx, (**D**) SOD, (**E**) MDA, and (**F**) protein carbonyl. Data are expressed as mean ± SEM based on 12 individual fish measurements per treatment, comprising four fish sampled from each of three replicate tanks. Different letters above the bars indicate significant differences among groups (*p* < 0.05).

After 60 days of salinity exposure, significant changes in antioxidant status were observed in grass carp gills. T-AOC was significantly decreased in the 8 g/L group (*p* < 0.05; Figure 3B). GPx and SOD activities were significantly reduced in both salinity-treated groups (*p* < 0.05; Figure 3C,D). MDA content increased significantly at 4 g/L but decreased significantly at 8 g/L relative to the 4 g/L group (*p* < 0.05; Figure 3E). However, GSH and protein carbonyl contents showed no significant changes among groups (*p* > 0.05; Figure 3A,F).

### 3.4. Metabolic Profile in Gills

**Figure 4:**
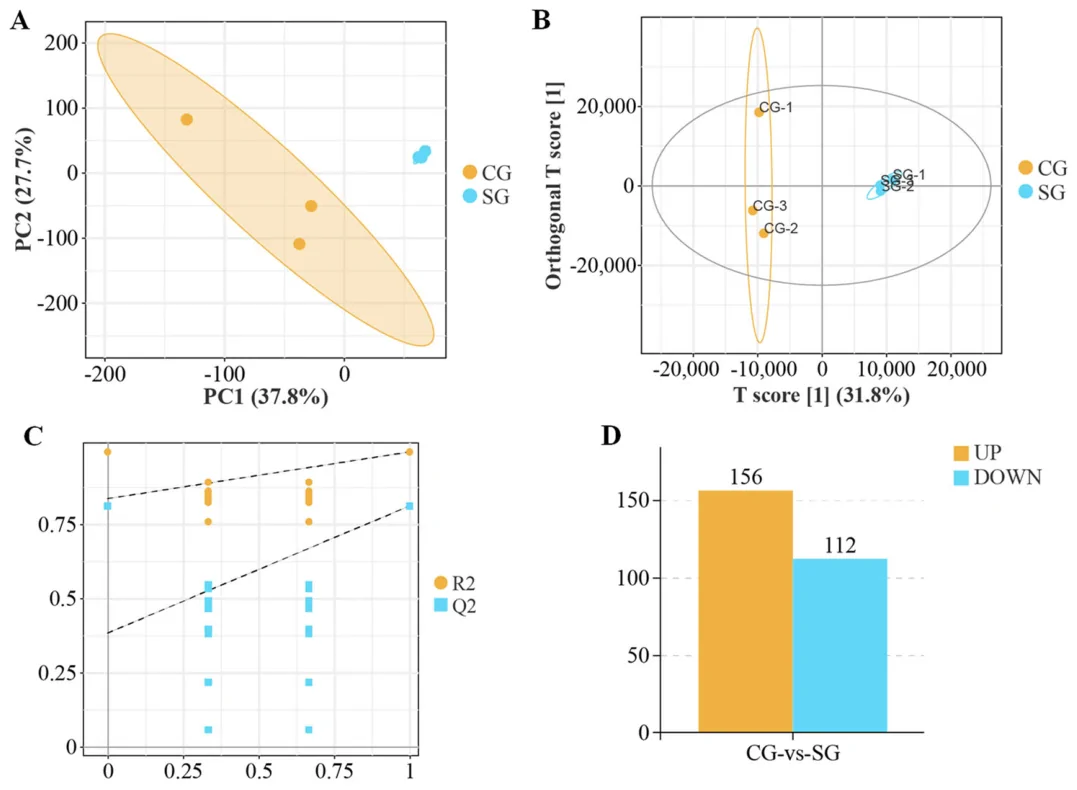
Metabolomic analysis of gill tissues in the CG and SG groups. (**A**) PCA score plot of samples from the CG and SG groups. (**B**) OPLS-DA score plot of samples from the CG and SG groups. (**C**) Permutation test of the OPLS-DA model. Orange and blue points represent the R^2^ and Q^2^ values of the permuted models, respectively, and the dashed lines indicate the corresponding linear regression trends. (**D**) Numbers of increased and decreased metabolites. CG, 0 g/L salinity; SG, 8 g/L salinity concentrations.

**Figure 5:**
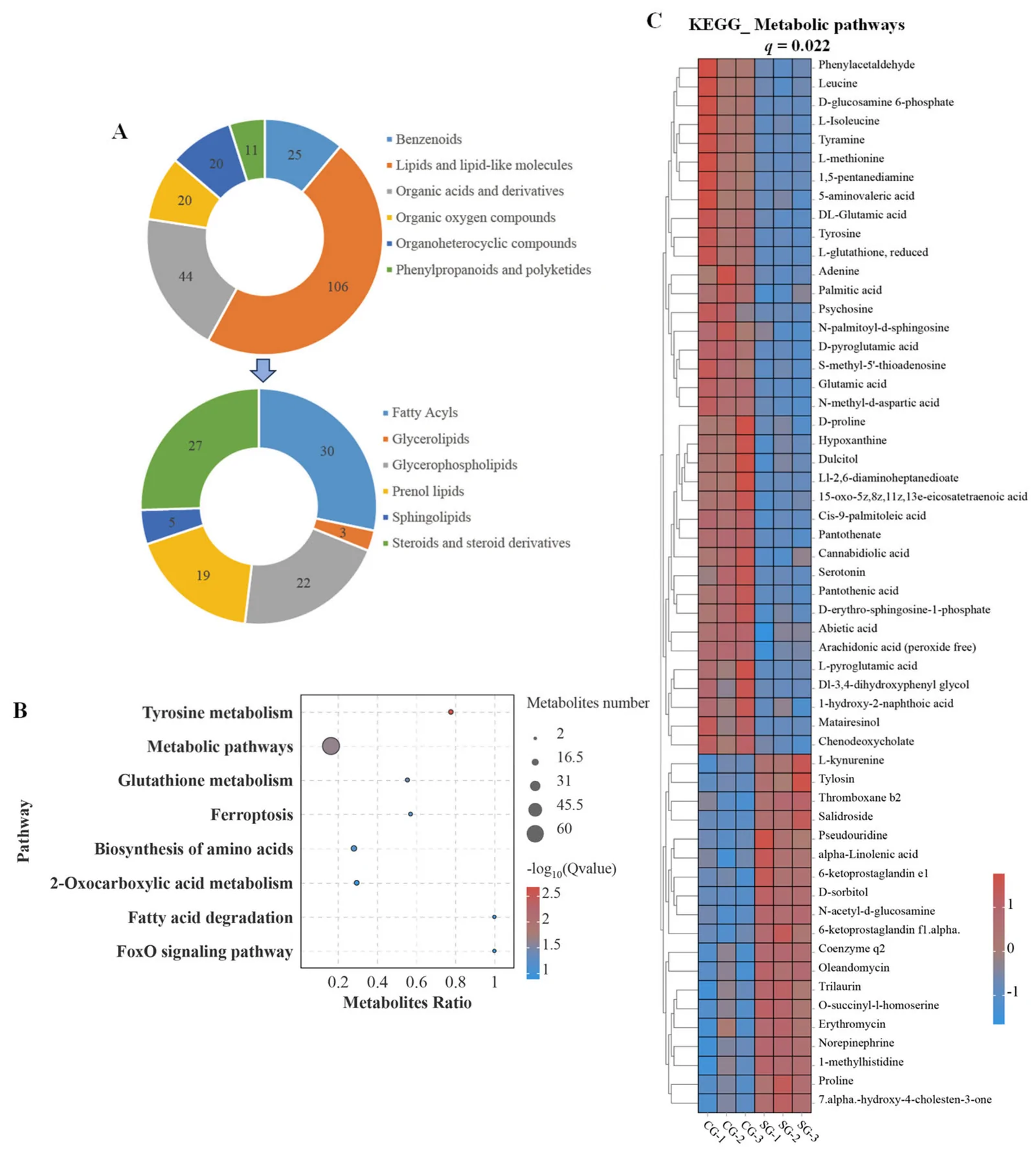
Differential metabolite profiles in the gills of grass carp after salinity exposure. (**A**) Classification and number of differential metabolites. (**B**) Major KEGG pathways significantly enriched by differential metabolites. (**C**) Relative abundance of differential metabolites involved in metabolic pathways. CG, 0 g/L salinity; SG, 8 g/L salinity.

Quality control (QC) analysis demonstrated the reliability of the metabolomic data, as indicated by the tight clustering of QC samples. A total of 3391 metabolites were annotated in gill tissues. PCA showed a clear separation between the CG and SG groups (Figure 4A). Consistent with the PCA results, OPLS-DA further revealed distinct metabolic profiles between the two groups (Figure 4B). In addition, cross-validation and permutation tests confirmed the robustness and reliability of the OPLS-DA model (Figure 4C). Compared with the CG group, 156 metabolites were significantly increased and 112 were significantly decreased in the SG group (Figure 4D). The differentially expressed metabolites (DEMs) were mainly classified into six major categories: lipids and lipid-like molecules (106), organic acids and derivatives (44), benzenoids (25), organic oxygen compounds (20), organoheterocyclic compounds (20), and phenylpropanoids and polyketides (11). Among these, lipids and lipid-like molecules were mainly represented by fatty acyls (30), steroids and steroid derivatives (27), glycerophospholipids (22), prenol lipids (19), sphingolipids (5), and glycerolipids (3) (Figure 5A). KEGG enrichment analysis showed that the DEMs were significantly enriched in several metabolism-related pathways, including tyrosine metabolism, glutathione metabolism, and metabolic pathways (Figure 5B). Within the metabolic pathways, 37 metabolites were decreased and 19 were increased after salinity exposure (Figure 5C).

### 3.5. Transcriptomic Analysis in Gills

**Figure 6:**
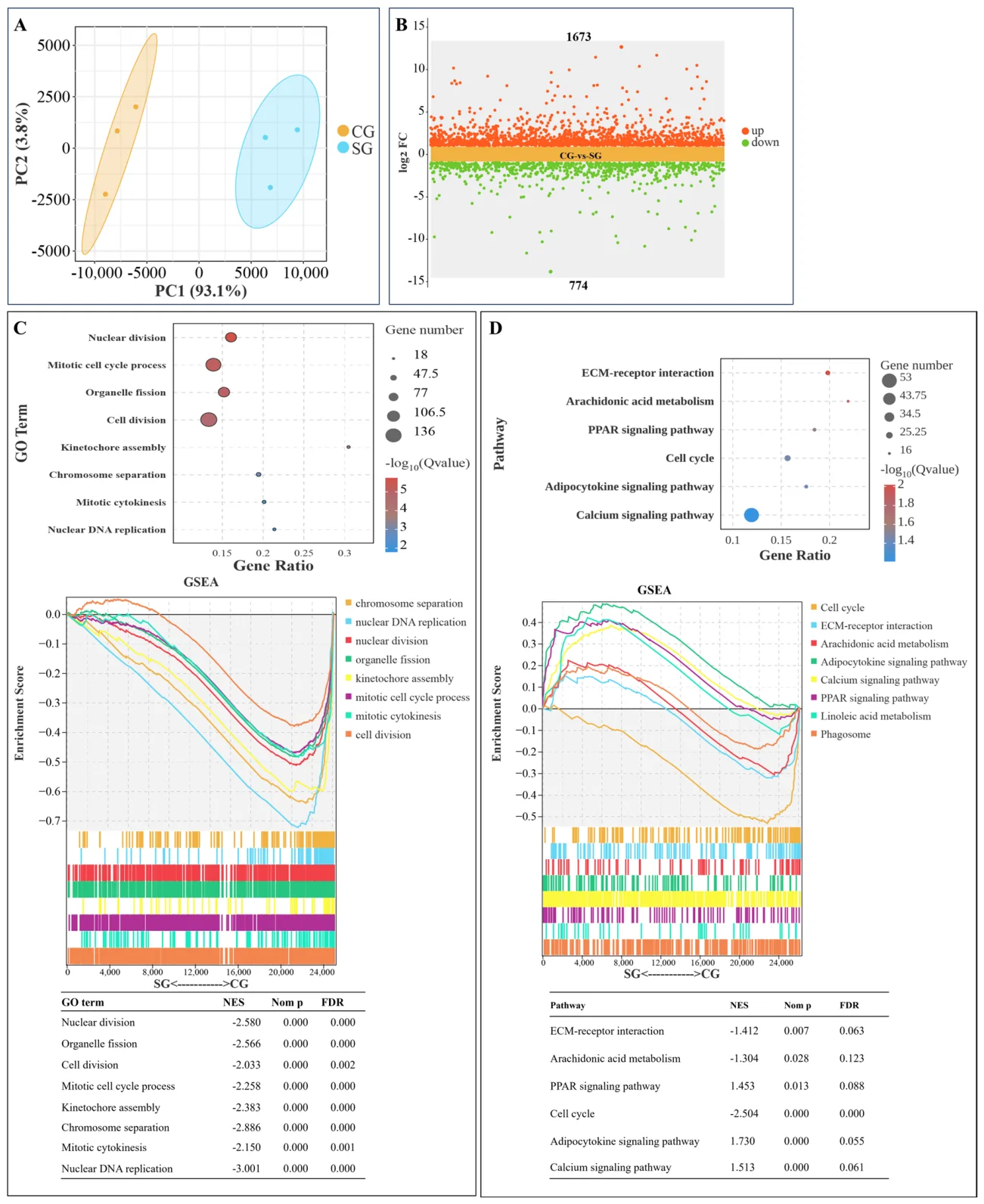
Transcriptomic analysis of gill tissues in grass carp after salinity exposure. (**A**) PCA score plot of samples from the control (CG) and salinity-treated (SG) groups. (**B**) Number of DEGs induced by salinity exposure. (**C**) GO enrichment analysis of DEGs (upper) and GSEA of selected GO terms (lower). (**D**) KEGG enrichment analysis of DEGs (upper) and GSEA of selected KEGG pathways (lower).

The Q20 and Q30 values were 97.95–98.32% and 93.37–95.03%, respectively, demonstrating high sequencing accuracy and reliability. The GC content ranged from 46.43% to 47.26%, with only minor variation among samples, suggesting good consistency in library construction. In addition, the total mapped rate ranged from 91.41% to 93.27%, indicating that most reads could be successfully aligned to the reference genome. Overall, these results demonstrate that the RNA-seq data were of high quality and can be reliably used for downstream analyses (Table S3). PCA revealed a clear separation between the CG and SG groups, indicating that salinity stress markedly altered the transcriptomic profile of gill tissue (Figure 6A). A total of 2447 DEGs were identified in the SG group compared with the CG group, including 1673 upregulated and 774 downregulated genes (Figure 6B). GO enrichment analysis revealed that the DEGs were significantly enriched in biological processes related to nuclear division, organelle fission, cell division, and mitotic cell cycle process (Figure 6C). Furthermore, GSEA results indicated a downward trend in these functional categories in the gills of grass carp following salinity exposure (Figure 6C).

KEGG enrichment analysis showed that the DEGs were significantly enriched in several pathways, including ECM–receptor interaction, arachidonic acid metabolism, the PPAR signaling pathway, cell cycle, the adipocytokine signaling pathway and the calcium signaling pathway (Figure 6D). GSEA indicated that ECM–receptor interaction, arachidonic acid metabolism and cell cycle showed a downward trend, whereas the PPAR signaling pathway, adipocytokine signaling pathway and calcium signaling pathway showed an upward trend in the gills of grass carp after salinity exposure (Figure 6D).

### 3.6. Alterations in Key Signaling Pathways

**Figure 7:**
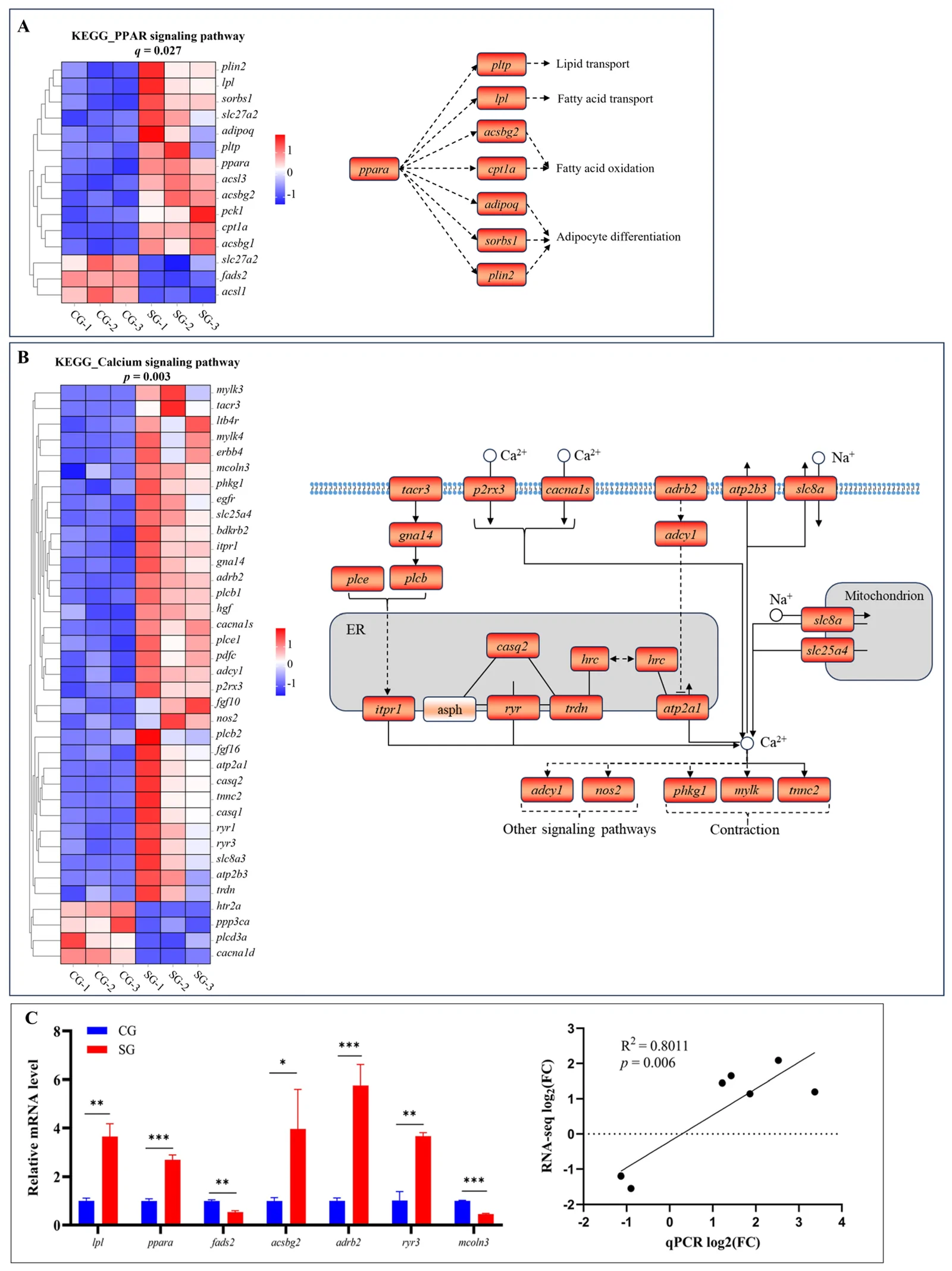
Changes in the PPAR signaling pathway and calcium signaling pathway in the gills of grass carp after salinity exposure. (**A**) DEGs in the PPAR signaling pathway. (**B**) DEGs in the calcium signaling pathway. Heatmap colors represent gene-wise (row-scaled) z-score-standardized expression values across the RNA-seq samples. (**C**) qPCR validation for RNA-seq. Data are presented as mean ± SEM (*n* = 3). CG, 0 g/L salinity; SG, 8 g/L salinity. Statistical significance is indicated by asterisks: * *p* < 0.05, ** *p* < 0.01 and *** *p* < 0.001.

Salinity exposure affected both the peroxisome proliferator-activated receptor (PPAR) signaling pathway and the calcium signaling pathway (Figure 7A,B). In the PPAR signaling pathway, 12 genes were upregulated and 3 genes were downregulated. These included peroxisome proliferator-activated receptor alpha (*ppara*), phospholipid transfer protein (*pltp*), lipoprotein lipase (*lpl*), long-chain-fatty-acid-CoA ligase ACSBG2-like (*acsbg2*), carnitine palmitoyltransferase 1A (*cpt1a*), adiponectin (*adipoq*), sorbin and SH3 domain containing 1 (*sorbs1*), and perilipin 2 (*plin2*), most of which showed increased expression. These transcriptional changes suggest that salinity stress may affect lipid transport, fatty acid transport, and fatty acid oxidation in grass carp gills.

In the calcium signaling pathway, 33 genes were upregulated and 4 genes were downregulated under salinity stress. Several genes involved in Ca^2+^ influx, endoplasmic reticulum Ca^2+^ release, and mitochondrial Ca^2+^ transport showed altered expression, including tachykinin receptor 3 (*tacr3*), purinergic receptor P2X 3 (*p2rx3*), calcium voltage-gated channel subunit alpha1 S (*cacna1s*), adrenergic receptor beta 2 (*adrb2*), ATPase plasma membrane Ca^2+^ transporting 3 (*atp2b3*), solute carrier family 8 member A (*slc8a*), inositol 1,4,5-trisphosphate receptor type 1 (*itpr1*), ATPase sarcoplasmic/endoplasmic reticulum Ca^2+^ transporting 1 (*atp2a1*), calsequestrin 2 (*casq2*), ryanodine receptor 1 (*ryr1*), ryanodine receptor 3 (*ryr3*), and solute carrier family 25 member 4 (*slc25a4*), with most showing an upward trend. Together with the positive GSEA enrichment of the calcium signaling pathway, these transcriptional changes are consistent with altered Ca^2+^ handling and increased involvement of Ca^2+^-associated regulatory processes under salinity stress. In addition, qPCR results showed significant agreement with the RNA-seq results for the selected genes (Figure 7C).

### 3.7. Integrated Transcriptomic and Metabolomic Analysis

**Figure 8:**
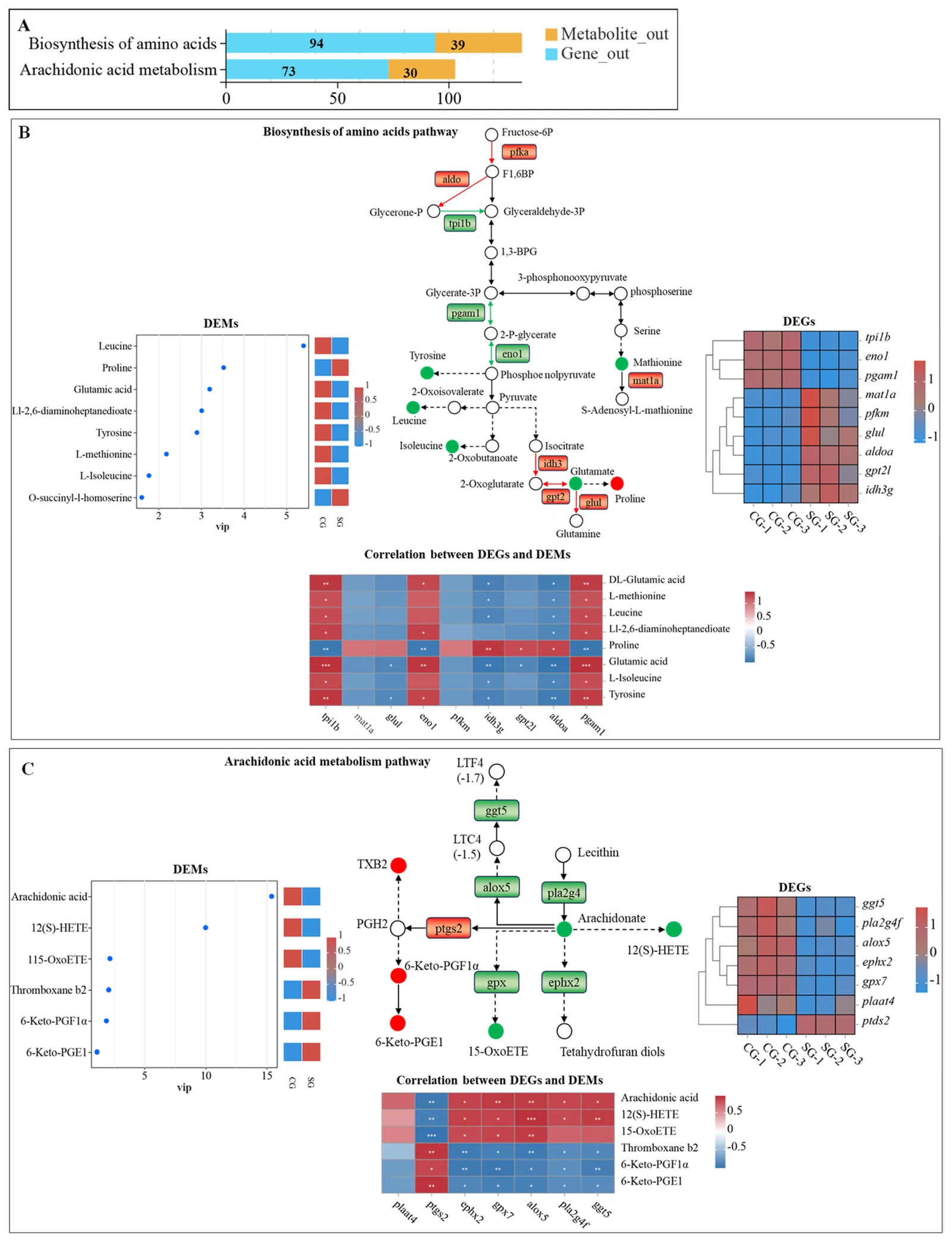
Integrated transcriptomic and metabolomic analysis in gills of grass carp after salinity exposure. (**A**) Genes and metabolites mapped to the amino-acid biosynthesis and arachidonic acid metabolism pathways. (**B**) DEGs and DEMs in biosynthesis of amino acids with their potential functional mechanisms. (**C**) DEGs and DEMs in arachidonic acid metabolism with their potential regulatory mechanisms. Red circles represent upregulated metabolites, while green circles indicate downregulated metabolites; red squares denote upregulated genes, and green squares signify downregulated genes.

Integrated transcriptomic and metabolomic analyses showed that amino-acid biosynthesis and arachidonic-acid metabolism were prominently associated with the gill response of grass carp to salinity stress. A total of 94 genes and 39 metabolites were mapped to the amino-acid biosynthesis pathway (Figure 8A). Of these, 9 genes and 8 metabolites met the predefined differential-expression and differential-abundance criteria, respectively. The 9 DEGs comprised 6 upregulated and 3 downregulated genes. Specifically, phosphofructokinase, alpha isoform (*pfka*), fructose-bisphosphate aldolase A (*aldoa*), glutamate-ammonia ligase (*glul*), glutamic-pyruvic transaminase 2-like (*gpt2l*), methionine adenosyltransferase 1A (*mat1a*), and isocitrate dehydrogenase 3 subunit gamma (*idh3g*) were upregulated, whereas triosephosphate isomerase 1b (*tpi1b*), phosphoglycerate mutase 1 (*pgam1*), and enolase 1 (*eno1*) were downregulated. Metabolomic analysis further revealed significant changes in 8 metabolites following salinity exposure. Among the metabolites, proline was increased, whereas leucine, glutamic acid, tyrosine, L-methionine, and L-isoleucine were decreased. These results indicate coordinated changes in gene expression and metabolite abundance in the amino acid biosynthesis pathway under salinity stress.

A total of 73 genes and 30 metabolites were mapped to the arachidonic-acid metabolism pathway. Of these, 7 genes and 6 metabolites met the predefined differential-expression and differential-abundance criteria, respectively. Specifically, gamma-glutamyl transferase 5 (*ggt5*), arachidonate 5-lipoxygenase (*alox5*), phospholipase A2 group IVA (*pla2g4*), and epoxide hydrolase 2 (*ephx2*) were downregulated under salinity stress. At the metabolite level, arachidonic acid, 12(S)-HETE, and 15-OxoETE were decreased, whereas thromboxane B2, 6-Keto-PGF1α, and 6-Keto-PGE1 were increased. These coordinated changes suggest a close association between transcriptional regulation and metabolite variation in the arachidonic acid metabolism pathway under salinity stress.

## 4. Discussion

The present study investigated the physiological and molecular responses of grass carp to salinity stress. Our results showed that salinity exposure affected gill morphology, ion regulation, antioxidant defense, and metabolic processes, indicating that grass carp initiate coordinated adaptive responses to cope with salinity stress.

### 4.1. Effect of Salinity on Gill Ion Regulation

The gill is a major site of ion regulation in fish, where ionocytes and associated transport proteins coordinate ion uptake, secretion, and epithelial homeostasis under changing environmental salinity. Previous studies have shown that freshwater fish can adjust their ion-regulatory strategies in response to salinity exposure, often accompanied by changes in systemic ion composition. For instance, common carp exhibited increased blood osmolality and elevated Na^+^ and Cl^−^ concentrations following salt exposure [42]. Similarly, Nile tilapia exposed to 15 ppt salinity for 10 days showed increased plasma levels of Na^+^, K^+^, and Ca^2+^ [43]. However, existing studies on the ionic effects of salinity have focused primarily on blood, whereas the tissue-specific ion responses, particularly in the gill, have rarely been explored. In the present study, long-term exposure to 8 g/L salinity resulted in a significant decrease in gill Na^+^ levels and an increase in K^+^, whereas no significant ionic shifts were observed at 4 g/L. This ion-specific pattern may be associated with salinity-induced alterations in branchial ionocytes and related ion transport processes, thereby affecting local ion distribution in gill tissue. Meanwhile, prolonged salinity exposure may also impair epithelial integrity and disrupt local ion homeostasis. Further studies are needed to clarify the mechanisms underlying these changes.

Na^+^/K^+^-ATPase (NKA) is one of the key enzymes involved in branchial ion regulation, and its response to salinity varies depending on species, salinity level, and exposure duration. In Nile tilapia, branchial NKA activity increased significantly after exposure to 2 and 8 ppt salinity [44]. In *Micropterus salmoides*, gill NKA activity initially increased and then decreased along a salinity gradient of 0, 5, 8, 9, and 10 ppt [45]. In contrast, in *Scatophagus argus*, NKA activity decreased under high salinity but increased under low salinity conditions [46]. A previous study also showed that acute exposure to 10 ppt salinity significantly increased gill NKA activity in grass carp [47]. In contrast, the present study showed that NKA activity was significantly reduced after 60 days of exposure to 8 g/L salinity. This difference between acute and chronic exposure suggests that NKA regulation in grass carp is dependent on the duration and intensity of salinity challenge. The reduced NKA activity observed after prolonged exposure may reflect a chronic adjustment of ion-transport processes and/or a decline in branchial ion-regulatory capacity under sustained salinity stress. Together with the concomitant decrease in gill Na^+^ concentration, these findings indicate that local Na^+^ handling and ion homeostasis were altered under long-term high-salinity exposure.

Notably, long-term salinity exposure decreased Ca^2+^ concentration in the gill while increasing Ca^2+^-ATPase activity, indicating that calcium regulation in the gill was markedly altered under these conditions. This pattern is partly consistent with previous findings in *Oreochromis niloticus*, in which gill Ca^2+^-ATPase activity increased at all salinities during chronic exposure [48]. Given the important role of Ca^2+^-ATPase in cellular Ca^2+^ transport and homeostasis, the elevated enzyme activity observed in the present study may have enhanced Ca^2+^ extrusion and/or redistribution, thereby contributing to the reduced Ca^2+^ level in gill tissue [49,50]. These findings suggest that, in addition to Na^+^ and K^+^ regulation, calcium handling in the gill is also responsive to prolonged salinity exposure. Branchial ion regulation involves multiple transport systems in addition to NKA and Ca^2+^-ATPase. NKCC and CFTR are important components of ion-secretory pathways, whereas NHE3 and NCC contribute to Na^+^ uptake in freshwater-adapted ionocytes [51,52]. V-type H^+^-ATPase also participates in ion uptake and acid–base regulation [53]. These transporters are differentially regulated during salinity acclimation and may therefore contribute to the altered ion profiles observed in the present study. Although these parameters were not measured in the present study, future work integrating transporter expression, protein localization and functional ion-flux measurements would provide a more comprehensive understanding of branchial osmoregulatory mechanisms in grass carp under long-term salinity exposure.

### 4.2. Salinity-Induced Alterations in Antioxidant Status

Salinity stress is known to induce the overproduction of reactive oxygen species (ROS), leading to oxidative damage. To maintain redox homeostasis, organisms activate a complex antioxidative defense system composed of both enzymatic components (e.g., SOD and CAT) and non-enzymatic antioxidants (e.g., GSH) [54]. As a significant environmental stressor, salinity can disrupt antioxidant metabolism in freshwater fish. Short-term or mild salinity exposure has been shown to enhance the activities of key antioxidative enzymes. For instance, SOD and GPx activities increased in *Oreochromis niloticus* exposed to 8 g/L salinity for 14 days [55]. Similarly, a significant rise in SOD and CAT activity was observed in the gills of silver carp following 48-h exposure to brackish water (6 g/L) [56]. In contrast, chronic or severe oxidative stress can deplete antioxidant reserves, impair the defense system, and ultimately lead to cell death or tissue damage [57]. In common carp, prolonged exposure to higher salinities (15 and 20 g/L) for 8 weeks resulted in decreased activities of SOD, CAT and GPx, and the authors suggested that sustained stress promotes excessive ROS generation, thereby inducing oxidative stress [58]. We hypothesize that elevated salinity may impair antioxidant capacity and increase oxidative challenge in the gill.

Long-term salinity exposure induced a non-linear change in MDA levels in grass carp gills, with an increase at 4 g/L followed by a decline at 8 g/L. Similar biphasic responses of MDA to salinity have been reported in other teleost tissues, suggesting that lipid peroxidation does not necessarily increase proportionally with salinity intensity or exposure duration. In *Pseudobagrus ussuriensis*, renal MDA levels first increased and subsequently decreased during prolonged salinity exposure [13]. The elevated MDA level at 4 g/L likely reflects enhanced lipid peroxidation during the initial oxidative response, whereas the decline observed at 8 g/L occurred concurrently with marked reductions in SOD, GPx and T-AOC, indicating a substantial weakening of antioxidant capacity. Under prolonged salinity challenge, sustained metabolic expenditure, lipid remodeling, and tissue injury may alter the abundance and composition of peroxidizable membrane lipid substrates, potentially limiting further MDA accumulation despite persistent redox disturbance [27,59]. This interpretation is supported, at least in part, by the metabolomic results, which revealed extensive alterations in lipid and lipid-like metabolites, including fatty acyls, glycerophospholipids, sphingolipids, and glycerolipids. Such remodeling of lipid metabolism may alter both the abundance and composition of peroxidizable substrates and consequently influence MDA production. Therefore, the reduced MDA level at 8 g/L may reflect a shift from active lipid peroxidation toward broader lipid metabolic remodeling and substrate limitation under prolonged salinity exposure, rather than a recovery of oxidative status. Nevertheless, because total lipid content, membrane lipid composition, and cell viability were not directly quantified, the relative contributions of lipid depletion and tissue damage remain to be clarified.

### 4.3. Modulation of Metabolic Functions in Response to Salinity

Salinity stress imposes additional energetic demands on fish for ion regulation, cellular maintenance, and tissue repair, thereby requiring metabolic reprogramming [5,60]. In the present study, integrated transcriptomic and metabolomic analyses demonstrated that long-term salinity exposure markedly altered metabolic pathways in the gills of grass carp. Among these changes, we focused particularly on two pathways: biosynthesis of amino acids and arachidonic acid metabolism.

After 60 days of exposure, the biosynthesis of amino acids pathway was altered, as indicated by the significant decreases in tyrosine, leucine, isoleucine, methionine, and glutamate in the 8 g/L group. These results suggest that prolonged salinity exposure disturbed amino acid homeostasis in the gill and may have reduced the metabolic capacity supporting amino acid biosynthesis. This interpretation is consistent with previous studies showing that salinity can affect amino acid metabolism in fish gills and other tissues [13]. It should be noted that steady-state metabolite abundance reflects the net balance among synthesis, degradation, transport, and utilization; therefore, the present metabolomic data alone cannot distinguish which of these processes primarily accounts for the observed decreases in amino acid levels. The decreases in leucine and isoleucine may indicate enhanced utilization of branched-chain amino acids during prolonged salinity acclimation. These amino acids are involved in both protein metabolism and energy metabolism [61], whereas glutamate is a key metabolic intermediate linking amino acid turnover with carbon and nitrogen metabolism [62]. Given that amino acids can be oxidized for ATP production and also used for macromolecular synthesis in osmoregulatory tissues during salinity adaptation, the depletion of these metabolites may reflect increased utilization and/or reduced biosynthetic availability under chronic salinity exposure [63].

The transcriptomic data provide additional context for these possible metabolic changes. PGAM1 and ENO1 are glycolysis-related enzymes that provide central carbon flux and metabolic intermediates for anabolic metabolism; therefore, their decrease may weaken glycolytic support for amino acid biosynthesis [64,65]. In parallel, the upregulation of *glul* may facilitate the conversion of glutamate to glutamine, which could partly explain the reduced glutamate level [66]. Moreover, increased expression of mat1a, a key enzyme catalyzing the conversion of methionine to S-adenosylmethionine, may enhance methionine utilization and thereby partly account for the reduced methionine level [67]. Taken together, these results suggest that long-term salinity exposure induces coordinated remodeling of amino acid metabolism in grass carp gills, likely reflecting a shift in metabolic resource allocation toward ionoregulatory and maintenance functions. In addition to amino acid metabolism, arachidonic acid metabolism was another pathway that showed a pronounced response to long-term salinity exposure in grass carp gills. In the present study, GSEA showed an overall reduced trend for this pathway, suggesting that lipid-derived stress signaling in the gill was altered under prolonged salinity exposure. In fish, arachidonic acid metabolism plays an important role in gill stress responses. Its bioactive metabolites are involved in local inflammatory and immune regulation and are closely associated with ion transport, osmoregulation, and oxidative stress in the gill epithelium [68].

In our study, the decreases in arachidonate, 12(S)-HETE, and 15-OxoETE, together with the downregulation of alox5, suggest reduced activity of lipoxygenase-related arachidonic acid metabolism [69]. Since arachidonic acid-derived eicosanoids are closely linked to oxidative stress, inflammatory signaling, and membrane homeostasis, these changes may reflect an adaptive attenuation of lipid-derived stress signaling during chronic salinity acclimation rather than the activation of a pronounced acute inflammatory response [70]. At the same time, the increases in TXB2 and 6-Keto-PGE1, both prostanoid-related metabolites, suggest that although parts of arachidonic acid metabolism appeared to be attenuated, specific prostanoid branches remained active or were selectively enhanced, possibly to support local vascular, epithelial, or homeostatic regulation in gill tissue [69,71]. Therefore, taken together with the GSEA result showing a downward trend, these findings suggest that long-term exposure to 8 g/L salinity induced a selective remodeling of arachidonic acid metabolism in grass carp gills. This response was characterized by attenuation of lipoxygenase- and epoxide-related branches, while specific prostanoid outputs appeared to be preserved or selectively enhanced, possibly contributing to local homeostatic regulation in gill tissue during salinity acclimation. However, prolonged suppression of arachidonic acid turnover could also alter membrane-associated signaling and epithelial repair capacity [72,73].

### 4.4. Underlying Mechanisms: Involvement of Key Signaling Pathways

In fish, PPARα is widely recognized as a key metabolic regulator involved in environmental stress responses. It participates in stress adaptation primarily by promoting fatty acid uptake and β-oxidation, remodeling energy metabolism, alleviating oxidative stress, and modulating inflammatory and immune responses [74,75,76]. Increased transcriptional involvement of the PPAR signaling pathway has been associated with metabolic adaptations involving lipid mobilization and fatty-acid utilization, which may contribute to meeting increased energetic demands under environmental stress [74,77]. In red tilapia, acute salinity stress has been shown to activate the PPAR signaling pathway in gill tissue [75]. Similarly, in largemouth bass, hyperosmotic stress was reported to enhance fatty acid β-oxidation and energy release through modulation of the PPAR and AMPK pathways and their associated hub gene [78]. Consistent with these previous findings, the present study showed that exposure to 8 g/L salinity upregulated 12 genes associated with the PPAR signaling pathway in the gills, including *pparα*, a key transcriptional regulator of the PPARα signaling axis. Together with the positive GSEA enrichment of the PPAR signaling pathway, these findings suggest that salinity stress may induce transcriptional remodeling of lipid metabolism in gill tissue, which could potentially facilitate lipid utilization and contribute to meeting the energetic demands associated with ion regulation and coordinated cellular regulation of ion homeostasis under saline conditions.

In addition to the PPAR signaling pathway, calcium signaling represents another critical mechanism underlying salinity adaptation in grass carp. Ca^2+^ signaling is widely considered an important regulatory system involved in environmental stress responses, contributing primarily to ion transport, osmotic adjustment, intracellular signal transduction, and cellular homeostasis [79]. As the major organ responsible for active Ca^2+^ uptake and Ca^2+^ homeostasis, the gill plays a central role in maintaining ionic balance in response to environmental change [49]. Previous studies have shown that alterations in environmental Ca^2+^ can rapidly trigger short-term transcriptional acclimation in fish gills, possibly accompanied by the regulation of genes involved in energy production and energy homeostasis [80]. Similarly, transcriptomic analysis in hybrid tilapia showed that short-term brackish water acclimation activated ion transport and calcium signaling in the gills [81]. Consistent with these previous findings, the present study showed that exposure to 8 g/L salinity increased the expression of 33 genes associated with the calcium signaling pathway in the gills, and GSEA showed positive enrichment of this pathway. These findings indicate transcriptional remodeling of Ca^2+^-associated processes in gill tissue in response to salinity-induced ionic disturbance. Given the concurrent alterations in ion concentrations, reduced Na^+^/K^+^-ATPase activity, and histological injury at 8 g/L, this transcriptional response may represent a compensatory attempt to restore ionic balance, although dysregulation, tissue injury, or changes in cellular composition may also contribute to the observed expression patterns.

Further analysis of the upregulated genes within the calcium signaling pathway revealed coordinated transcriptional changes in genes associated with intracellular Ca^2+^ handling. Specifically, *itpr1*, *ryr*, *casq2*, *trdn*, *hrc*, and *atp2a1* were significantly upregulated following salinity exposure [82]. ITPR1 and RYR are associated with Ca^2+^ release from intracellular stores [83,84], whereas CASQ2, TRDN, and HRC participate in Ca^2+^ storage and regulation of Ca^2+^ release complexes [85,86]. In addition, ATP2A1 encodes a SERCA Ca^2+^ pump involved in Ca^2+^ reuptake into the ER/SR [87,88]. These transcriptional changes, together with the observed alterations in gill Ca^2+^ concentration and Ca^2+^-ATPase activity, suggest that prolonged salinity exposure affects Ca^2+^ handling and calcium-related signaling processes in grass carp gills. Further functional studies are required to determine whether these transcriptional responses translate into changes in Ca^2+^ signaling activity. One limitation of the present study is that circulating biochemical and endocrine parameters were not evaluated. Therefore, although the present transcriptomic and metabolomic results characterize metabolic changes occurring in the gill, they do not allow us to determine the tissue origin of these metabolites or their transport between organs. Future studies integrating plasma biochemical indices with parallel analyses of the liver and gills are needed to better characterize whole-organism metabolic allocation and inter-organ coordination under chronic salinity stress.

## 5. Conclusions

In conclusion, chronic salinity exposure significantly affected the structure and function of grass carp gills, with responses differing among salinity treatments. Moderate salinity induced limited adaptive responses, whereas high salinity caused clear histopathological damage, disturbed ionic balance, and weakened antioxidant capacity. Multi-omics analyses revealed that salinity stress markedly altered transcriptional and metabolic profiles, particularly in pathways associated with amino acid biosynthesis, arachidonic acid metabolism, PPAR signaling, and calcium signaling. These changes indicate that grass carp respond to salinity stress through coordinated changes in ion homeostasis, antioxidant defense, lipid metabolism, and cellular signaling. Overall, this study provides comprehensive evidence that the gill is a major target organ in salinity stress response and identifies key molecular pathways involved in the adaptation of grass carp to high salinity environments. These findings improve our understanding of salinity adaptation in freshwater fish and help evaluate salinity tolerance and optimize aquaculture management strategies for grass carp.

## Data Availability

The raw transcriptomic sequencing data have been deposited in the Genome Sequence Archive (GSA) of the China National Center for Bioinformation (CRA038997), and other data can be obtained by contacting the authors.

## Supplementary Materials

The following supporting information can be downloaded at: https://www.mdpi.com/article/10.3390/antiox15091070/s1, Method S1. Na^+^/K^+^-ATPase (NKA) activity assay; Method S2. Ca^2+^-ATPase activity assay; Method S3. Protein carbonyl content assay; Method S4. Detailed LC–MS/MS conditions for untargeted metabolomics; Method S5. Data Processing and Metabolite Annotation; Method S6. RT-qPCR validation of RNA-seq data; Table S1. Effect of salinity stress on growth performance of grass carp after 60 days of exposure; Table S2. Primer sequences used for quantitative real-time PCR; Table S3. Quality assessment of RNA-seq data.

## Abbreviations

The following abbreviations are used in this manuscript:

Na^+^/K^+^-ATPase	Sodium/potassium-transporting ATPase
Ca^2+^-ATPase	Calcium-transporting ATPase
PPAR	Peroxisome proliferator-activated receptor
TCA	Tricarboxylic acid
ATP	Adenosine triphosphate
MS-222	Tricaine methanesulfonate
H&E	Hematoxylin and eosin
SOD	Superoxide dismutase
GSH	Glutathione
T-AOC	Total antioxidant capacity
GPx	Glutathione peroxidase
MDA	Malondialdehyde
WST-1	Water-soluble tetrazolium salt-1
DTNB	5,5′-Dithiobis-(2-nitrobenzoic acid)
TBA	Thiobarbituric acid
BCA	Bicinchoninic acid
PCA	Principal component analysis
QC	Quality control
FDR	False discovery rate
OPLS-DA	Orthogonal partial least squares-discriminant analysis
GO	Gene Ontology
KEGG	Kyoto Encyclopedia of Genes and Genomes
RSEM	RNA-Seq by Expectation-Maximization
qPCR	Quantitative real-time polymerase chain reaction
UHPLC	Ultra-high-performance liquid chromatography
LC–MS	Liquid chromatography–mass spectrometry
DEGs	Differentially expressed genes
GSEA	Gene Set Enrichment Analysis
GSA	Genome Sequence Archive
DEMs	Differentially expressed metabolites
PGAM1	Phosphoglycerate mutase 1
HETE	Hydroxyeicosatetraenoic acid
TXB2	Thromboxane B2
ITPR1	Inositol 1,4,5-trisphosphate receptor type 1
RYR	Ryanodine receptor
HRC	Histidine-rich calcium-binding protein
SERCA	Sarco/endoplasmic reticulum Ca^2+^-ATPase
