# Supplementary material for: Integrative Multi-Omics Analysis of Gill Responses to Long-Term Salinity Stress in Grass Carp (Ctenopharyngodon idella)
Source: Antioxidants (Basel). 2026 Aug 26;15(9):1070. doi: 10.3390/antiox15091070 (PMC13603754; doi:10.3390/antiox15091070)
Supplement: Supplementary file 1 [file antioxidants-15-01070-s001.zip › antioxidants-4460195-supplementary.pdf]

Method S1. Na^+/K^+-ATPase (NKA) activity assay

Gill Na^+/K^+-ATPase (NKA) activity was determined using an ouabain-sensitive enzyme-coupled spectrophotometric assay according to established processing with minor modifications. Briefly, frozen gill tissues were thawed on ice, weighed, and homogenized at a ratio of 1:10 (w/v) in ice-cold SEID buffer containing 150 mM sucrose, 10 mM EDTA, 50 mM imidazole, and 0.5% sodium deoxycholate (pH 7.5). The homogenates were centrifuged at 2,000 × g for 5 min at 4 °C, and the resulting supernatants were collected for enzyme activity determination. The reaction mixture contained 30 mM imidazole, 45 mM NaCl, 15 mM KCl, 3 mM $MgCl_2$, 0.4 mM KCN, 1 mM ATP, 0.2 mM NADH, 3 U/mL pyruvate kinase, 2 U/mL lactate dehydrogenase, 0.1 mM fructose-1,6-bisphosphate, and 2 mM phosphoenolpyruvate (pH 9.0). For each sample, 5 μL of supernatant was added to 200 μL of reaction mixture in either the absence or presence of 2 mM ouabain, a specific inhibitor of NKA. Reactions were performed in quadruplicate in 96-well microplates, and NADH oxidation was monitored by recording the decrease in absorbance at 340 nm at 1-min intervals for 15 min using a microplate reader. NKA activity was calculated as the difference between total ATPase activity measured in the absence of ouabain and ouabain-insensitive ATPase activity measured in the presence of ouabain and was normalized to sample protein content.

Method S2. Ca^{2+}-ATPase activity assay

Gill Ca^{2+}-ATPase activity was determined by measuring the liberation of inorganic phosphate (Pi) from ATP following the established method with minor modifications. Briefly, gill tissues were homogenized on ice in a buffer containing 100 mM Tris-HCl, 2 mM EDTA, and 5 mM $MgCl_2$ (pH 7.75). The homogenates were centrifuged at 12,000 × g for 5 min at 4 °C, and the resulting supernatants were used for enzyme activity determination. The reaction was conducted in a final volume of 0.5 mL containing 80 mM NaCl, 5 mM $MgCl_2$, 3 mM ATP, 20 mM Tris-HCl (pH 7.4), 0.5 mM $CaCl_2$, and 1 mM ouabain. Ouabain was included to inhibit Na^+/K^+-ATPase activity. The reaction was initiated by the addition of tissue homogenate and incubated at 30 °C for 30 min, after which the reaction was terminated by adding 0.2 mL of 20% trichloroacetic acid. Following centrifugation to remove precipitated proteins, the amount of released Pi was determined colorimetrically using the ammonium molybdate–ascorbic acid method. Briefly, 0.5 mL of the reaction supernatant was mixed with 1.0 mL of a 1:6 (v/v) mixture of 10% ascorbic acid and 0.42% ammonium molybdate prepared in 0.5 mM H_2SO_4 and incubated at 30 °C for 30 min. Absorbance was measured at 620 nm using a spectrophotometer, and Pi concentrations were calculated from a KH_2PO_4 standard curve. Protein concentrations of the homogenates were determined using the Bradford method with bovine serum albumin as the standard, and Ca^{2+}-ATPase activity was normalized to protein content.

Method S3. Protein carbonyl content assay

Protein carbonyl content was determined separately using the 2,4-dinitrophenylhydrazine (DNPH) derivatization method. Briefly, gill tissues were homogenized on ice in ice-cold phosphate buffer (50 mM, pH 7.4) at a tissue-to-buffer ratio of 1:9 (w/v), and the homogenates were centrifuged at 10,000 × g for 10 min at 4 °C. The resulting supernatants were collected for analysis. An aliquot of each sample was mixed with an equal volume of 10 mM DNPH prepared in 2 M HCl, while a corresponding blank was treated with 2 M HCl without DNPH. Samples were incubated for 1 h at room temperature in the dark with intermittent mixing. Proteins were subsequently precipitated by adding ice-cold 20% trichloroacetic acid and centrifuged at 10,000 × g for 10 min. The resulting pellets were washed three times with ethanol/ethyl acetate (1:1, v/v) to remove excess DNPH and then dissolved in 6 M guanidine hydrochloride. After centrifugation to remove insoluble material, the absorbance of the supernatant was measured at 370 nm against the corresponding HCl-treated blank. Protein carbonyl content was calculated using a molar extinction coefficient of 22,000 M^{-1} cm^{-1} and normalized to the protein concentration of each sample.

Method S4. Detailed LC–MS/MS conditions for untargeted metabolomics

Untargeted metabolomic analysis was performed using an Agilent 1290 Infinity LC ultra-high-performance liquid chromatography (UHPLC) system (Agilent Technologies, USA) coupled to an AB Sciex TripleTOF 6600 quadrupole time-of-flight mass spectrometer (AB Sciex Pte. Ltd., USA). Chromatographic separation was performed using an ACQUITY UPLC BEH Amide column (2.1 × 100 mm, 1.7 μm; Waters Corporation, USA) under hydrophilic interaction liquid chromatography (HILIC) conditions. The column temperature was maintained at 25 °C, the flow rate was 0.5 mL min^{-1}, and the injection volume was 2 μL. Mobile phase A consisted of water containing 25 mM ammonium acetate and 25 mM ammonium hydroxide, whereas mobile phase B consisted of acetonitrile. The gradient program was as follows: 0–0.5 min, 95% B; 0.5–5.0 min, a linear decrease from 95% to 65% B; 5.0–5.5 min, a linear decrease from 65% to 40% B; 5.5–6.0 min, a linear increase from 40% to 95% B; and 6.0–8.0 min, 95% B. Samples were maintained at 4 °C in the autosampler and analyzed in a randomized injection order. Pooled quality-control (QC) samples were interspersed throughout the analytical sequence to monitor instrument stability and analytical reproducibility. Mass-spectrometric data were acquired using an electrospray ionization (ESI) source in both positive- and negative-ion modes. The ion-source parameters were set as follows: ion source gas 1 (Gas1), 60; ion source gas 2 (Gas2), 60; curtain gas (CUR), 30 psi; source temperature, 600 °C; and ion spray voltage floating (ISVF), +5500 V in positive-ion mode and −5500 V in negative-ion mode. Full-scan MS spectra were acquired over an m/z range of 60–1000 with an accumulation time of 0.20 s per spectrum. MS/MS spectra were acquired over an m/z range of 25–1000 with an accumulation time of 0.05 s per spectrum. Data-dependent MS/MS acquisition was performed using information-dependent acquisition (IDA) based on ion-intensity selection.

Method S5. Data Processing and Metabolite Annotation

The raw data were converted to mzML format using ProteoWizard and subsequently processed using XCMS for peak detection, retention-time correction, peak alignment, and feature extraction. For peak picking using the centWave algorithm, the mass tolerance was set to 10 ppm, the peak-width range was 10–60 s, and the prefilter parameter was set to c (10, 100). For peak grouping, the bandwidth (bw), m/z width (mzwid), and minimum fraction (minfrac) were set to 5, 0.025, and 0.5, respectively. CAMERA was used to annotate isotopic peaks and adducts. Features with more than 50% missing values were removed, the remaining missing values were imputed using the K-nearest-neighbor (KNN) method, and features with a relative standard deviation (RSD) greater than 50% were excluded from subsequent analyses. Pooled quality-control (QC) samples were analyzed throughout the analytical sequence to monitor instrumental stability and overall data quality. Metabolite annotation was performed by comparing accurate m/z values and MS/MS spectra with an in-house spectral database established using available authentic standards, with a mass-error tolerance of <10 ppm for accurate-mass matching. Annotated metabolites were subsequently mapped to corresponding KEGG compound IDs for pathway annotation and enrichment analysis. Z-score transformation was applied where indicated for visualization of metabolite abundance patterns, including clustering heatmaps and Z-score plots.

Method S6. RT-qPCR validation of RNA-seq data

Gene-specific primers were designed using NCBI Primer-BLAST based on the corresponding grass carp RefSeq sequences. The primer sequences and corresponding NCBI RefSeq accession numbers are provided in Table S2. The same total RNA preparations used for transcriptome sequencing were used for RT-qPCR validation. cDNA was synthesized using the PrimeScript™ RT Reagent Kit with gDNA Eraser (Perfect Real Time; Takara Bio, Japan; RR047A) according to the manufacturer's instructions. Total RNA 1 μg was used in each 20-μL reverse-transcription reaction. Residual genomic DNA was removed by incubation with gDNA Eraser at 42 °C for 2 min. Reverse transcription was subsequently performed at 37 °C for 15 min, followed by enzyme inactivation at 85 °C for 5 s. RT-qPCR was performed on a C1000 Touch Thermal Cycler (BIO-RAD, USA) using TB Green® Premix Ex Taq™ II (Tli RNaseH Plus; Takara Bio, Japan; RR820A). Each reaction was conducted in a total volume of 20 μL, with forward and reverse primers each at a final concentration of 0.4 μM. The thermal cycling program consisted of initial denaturation at 95 °C for 30 s, followed by 40 cycles of denaturation at 95 °C for 5 s and annealing/extension at 60 °C for 30 s. Each biological sample was analyzed in triplicate technical reactions. Relative expression levels were calculated using the 2^−ΔΔCt method, with *β-actin* used as reference genes and the control group used as the calibrator.

Table S1. Effect of salinity stress on growth performance of grass carp after 60 days of exposure

	0 g/L	4 g/L	8 g/L
Initial weight (g)	103.08 ± 0.70	105.17 ± 1.71	102.31 ± 0.91
Terminal weight (g)	261.55 ± 18.06^{a}	248.34 ± 15.65^{a}	133.73 ± 9.91^{b}
Number of deaths	0	0	1

Note: Within the same row, values with different superscript letters indicate significant differences among treatment groups ($p < 0.05$).

Table S2. Primer sequences used for quantitative real-time PCR

Gene	NCBI Ref-Seq accession No.	Forward primer（5′–3′）	Reverse primer（5′–3′）
lpl	XM_051909470.1	TCCCTTACGTTATGCCTGCTT	TCACCTCCACGGGAAAGGTA
ppara	XM_051883407.1	CTCCACTGCGTTAGATGCAC	GGTGAACTCTTCCTGTCCCG
fads2	XM_051883101.1	GTGGCGCTCGAATGATCTGT	TGCTGCTTACTTTGCACTTCTC
acsbg2	XM_051893990.1	ACTGTAGCACTCCATGACTCC	GCCGCTTCATCGTCTGTATC
adrb2	XM_051860607.1	TAGCCTGGGAGCCACATTTA	GTGTGTTTCCCATGACCTCC
ryr3	XM_051874141.1	TGAAGATGAGGTGGTGCTGC	CTCCTTGTGCTGCTCCTTCA
mcoln3	XM_051877952.1	TCATCACGCTCATCACGGAC	ATCAGAGCCGCATATCCTGTG
β-actin (actb2)	XM_051886219.1	CCAAAGCCAACAGGGAAAAG	GCACAGTGTGGGTGACACCA

Table S3. Quality assessment of RNA-seq data

Samples	Raw bases (bp)	Clean bases (bp)	Q20 (%)	Q30 (%)	GC (%)	Total mapped (%)
CG1	6,661,774,200	6,605,021,883	98.09	93.80	47.13	93.27
CG2	6,522,131,700	6,456,424,381	98.06	93.63	47.17	93.14
CG3	6,695,031,900	6,640,765,148	98.20	94.04	47.26	93.14
SG1	6,957,304,800	6,899,281,568	97.95	93.37	46.43	92.89
SG2	6,619,440,300	6,556,864,187	98.10	93.75	46.45	92.93
SG3	6,571,086,300	6,512,760,376	98.32	95.03	47.05	91.41

Note: Q20 and Q30 represent the percentages of bases with Phred quality scores ≥20 and ≥30, respectively. GC content represents the percentage of guanine (G) and cytosine (C) bases in the clean sequencing data.
